# Th1-Like Treg Cells Are Increased But Deficient in Function in Rheumatoid Arthritis

**DOI:** 10.3389/fimmu.2022.863753

**Published:** 2022-05-04

**Authors:** Rui Zhang, Jinlin Miao, Kui Zhang, Bei Zhang, Xing Luo, Haoyang Sun, Zhaohui Zheng, Ping Zhu

**Affiliations:** ^1^ Department of Clinical Immunology, Chinese People's Liberation Army. (PLA) Specialized Research Institute of Rheumatoid & Immunology, Xijing Hospital, The Fourth Military Medical University, Xi’an, China; ^2^ Department of Special Service Convalescence, Air Force Healthcare Center for Special Services, Hangzhou, China; ^3^ National Translational Science Center for Molecular Medicine & Department of Cell Biology, The Fourth Military Medical University, Xi’an, China

**Keywords:** rheumatoid arthritis, regulatory T cell (Treg), effector T cell (Teff), Treg subset, synovial fluid

## Abstract

**Objectives:**

This study aimed to investigate the changes in quantity and function of T helper (Th)-like T regulatory (Treg) cell subsets in peripheral blood (PB) and synovial fluid (SF) of rheumatoid arthritis (RA) patients and to understand their relationship with disease activity.

**Methods:**

A total of 86 RA patients and 76 gender and age-matched healthy controls (HC) were enrolled in this study. Th-like Treg frequency and function were determined using flow cytometry. The inhibitory function of Th-like Treg cells was detected using an *in vitro* co-culture suppression assay.

**Results:**

The proportion and absolute number of Th1-like Treg cells from RA PB and RA SF were significantly higher than those of HC PB. In RA SF, the proportions of Treg cells and Th1-like Treg cells were significantly lower in the elevated erythrocyte sedimentation rate or the C-Reactive Protein group, and in the positive groups of anti-CCP antibody and anti-MCV antibody. Additionally, the proportions of Treg cells and Th1-like Treg cells from RA SF were negatively correlated with disease activity. However, the expression levels of CD73 and TGF-β1 in Th1-like Treg cells were decreased, and these Treg cells could not effectively inhibit the proliferation of effector T (Teff) cells.

**Conclusion:**

Our data indicate that Th1-like Treg cells are the predominant Treg cell subset in RA SF, but their suppressive function is defective. Improving the function of Th1-like Treg cells may control inflammation in joints and provide new strategies for Treg-targeted therapies in RA.

## Introduction

Rheumatoid arthritis (RA) is a chronic systemic autoimmune disease characterized by joint inflammation and multiple systemic lesions (1). However, up to now, the etiology and pathogenesis of RA have not been fully elucidated. Studies have shown that RA is caused by complex interactions between various immune cells, such as T cells, macrophages, and B cells (2, 3). The imbalance between proinflammatory T helper cells (Th cells) and anti-inflammatory regulatory T cells (Treg cells) plays a vital role in the pathogenesis and development of RA (4, 5).

Treg cells are a subset of CD4^+^ T cells that highly express CD25 and forkhead box protein P3 (Foxp3) and play an important role in maintaining immune system homeostasis (6). The number or functional deficiency of Treg cells is closely related to the incidence and development of many diseases, especially autoimmunity diseases (7). The role of Treg cells in the pathogenesis of RA has attracted much attention, but studies have reached different conclusions about the number and function of Treg cells in RA. Changes in the proportion and number of Treg cells in the peripheral blood (PB) of RA patients remain controversial (8–10), although the proportion of Treg cells increases in the SF of RA patients (10, 11). However, there is still controversy with regard to whether the inhibitory function of Treg cells in RA is defective. One study indicated that although Treg cells in RA can produce inflammatory cytokines, their inhibition of T cell proliferation and interleukin (IL)-17 production is enhanced (12). Nie H et al. revealed reduced inhibition of Treg cells in RA (13), while Hong X et al. showed that the function of Treg cells is not defective (14). Thus, no conclusion has yet been reached on the functional changes of Treg cells in RA, but it has been proposed that Treg heterogeneity in phenotype and function could play an important role in RA.

Until recently, there has been no agreement on a method to systematically and comprehensively classify Treg subsets. Based on their origin, Treg cells are divided into two groups: thymus-derived Treg (tTreg) cells and peripheral Treg (pTreg) cells. According to the expression of CD45RA and Foxp3, Treg cells can be further classified into three subsets: CD45RA^+^Foxp3^lo^ resting Treg (rTreg) cells, CD45RA^−^Foxp3^hi^ activated Treg (aTreg) cells, and cytokine-secreting CD45RA^−^Foxp3^lo^ non-suppressive T (non-Treg) cells, most of which are not Treg cells (15). Recently, Treg cells that are phenotypically similar to effector Th cells, referred to as Th-like Treg cells, have been largely studied in rodents and humans (16, 17). Th-like Treg cells express the same chemokine receptors as the corresponding T cells and play a regulatory or proinflammatory role during an immune response (18–21). Th1-like Treg cells express C-X-C motif chemokine receptor 3 (CXCR3) in a T-bet-dependent manner, allowing them to migrate to sites of type 1 inflammation and block Th1 cell responses (22–24). Both Th2-like Treg cells and Th17-like Treg cells express CCR4, while Th17-like Treg cells also express CCR6. Th17-like and Th2-like Treg cells can also express transcription factor RAR-related orphan receptor γt (RORγt) (21) and GATA-3 (25), allowing them to suppress Th17 and Th2 cell responses, respectively. Similar to Th22 cells, Th22-like Treg cells express the cutaneous lymphocyte antigen (CLA), along with the chemokine receptors CCR4, CCR6, and CCR10 (16). However, in some autoimmune diseases and tumors, Th-like Treg cells secrete pro-inflammatory cytokines and have reduced inhibitory function (26, 27). Even with this knowledge, the role of Th-like Treg cells in these diseases needs to be further studied.

In this study, we examined the changes in the number and function of Th-like Treg cell subsets in RA patients, laying a foundation for further exploring the role of Treg cell subsets in the pathogenesis of RA and developing targeted therapy for specific Treg subsets in RA.

## Materials and Methods

### Patients and Controls

This study included 86 RA patients (71 women and 15 men; mean age of 50.10 ± 12.66 years) recruited from the Clinical Immunology Department of Xijing Hospital, Shannxi, China, from November 2018 to January 2020. Synovial fluid (SF) was collected from 42 patients. All patients met the ACR/EULAR RA classification and the following criteria for treatment: (1) untreated or in drug withdrawal for more than 3 months; or (2) no change in treatment plan in the previous three months without high-dose steroids or immunosuppressants. The study also included 76 sex and age-matched (60 women and 16 men; mean age of 49.19 ± 12.10 years) healthy controls (HC) during the same period. Clinical data from enrolled patients were collected, namely, gender, age, course of disease, number of swollen and tender joints, and disease activity score in 28 joints (DAS28). Laboratory examination data were obtained at the same time of sample collection. The demographic and clinical characteristics of patients are summarized in **Table 1**. The Ethics Committee of Xijing Hospital approved the study, and all participating subjects signed informed consent.

**Table 1:** Demographic and clinical characteristic of subjects.

Parameter	RA (n = 86)	SF (n = 42)	HC (n = 76)
Age, (years, mean ± SD)	50.10 ± 12.66	51.90 ± 11.32	49.19 ± 12.10
Female/male	71/15	32/10	60/16
Disease duration, month (IQR)	72 (24–156)	96 (24–171)	NA
DAS28 (ESR, mean ± SD)	4.37 ± 1.52	4.63 ± 1.53	NA
DAS28 (CRP, mean ± SD)	4.10 ± 1.46	4.27 ± 1.38	NA
ESR (mm/h, IQR)	25 (11–44)	31 (16–67)	NA
CRP (mg/dl, IQR)	0.91 (0.31–3.85)	1.85 (0.45–7.38)	NA
RF positivity, n (%)	56 (65.11%)	27 (64.29%)	NA
Anti-CCP positivity, n (%)	50 (58.13%)	31 (73.81%)	NA
Anti-MCV positivity, n (%)	51 (59.30%)	30 (71.43%)	NA
AKA positivity, n (%)	35 (40.69%)	24 (57.14%)	NA
**Medication**			
DMARDs, n (%)	54 (62.79%)	16 (38.10%)	NA
Glucocorticoids, n (%)	13 (15.11%)	8 (19.05%)	NA
Anti-TNF therapy, n (%)	4 (4.65%)	4 (9.52%)	NA

SD, standard deviation; ESR, erythrocyte sedimentation rate; CRP, C-Reactive Protein; RF, rheumatoid factor; anti-CCP antibody, anti-cyclic citrullinated peptides antibody; anti-MCV antibody, anti-mutated citrullinated vimentin antibody; AKA, anti-keratin antibody; DMARDs, disease-modifying antirheumatic drugs; TNF, tumor necrosis factor; NA, not applicable.

### Detection of Th-Like Treg Cells Using Flow Cytometry

The following monoclonal antibodies (Abs) were used for surface phenotype and intracellular cytokine staining: peridinin chlorophyll protein (PerCP)/Cyanine5.5 anti-human CD4, fluorescein isothiocyanate anti-human CD45RA, Alexa Fluor 700 anti-human CD183 (CXCR3), Brilliant Violet 510 anti-human CD196 (CCR6), allophycocyanin (APC) anti-human CCR10 (all from Biolegend), Brilliant Violet 421 anti-human CD25, phycoerythrin (PE)-CF594 mouse anti-human CD194 (CCR4) (all from BD Biosciences), and PE anti-human Foxp3 (from eBioscience). Precision Count Beads (from Biolegend) were used to obtain absolute cell counts. Cells were acquired and analyzed using the CytoFLEX S flow cytometer (Beckman) and CytExpert software.

### Phenotype Analysis of Treg Cell Subsets

Treg cell subsets were evaluated for the expression of CTLA-4, CD73, CD39, IL-10, and tumor growth factor-β1 (TGF-β1). Cells were stained with Abs including Brilliant Violet 605 anti-human CTLA-4, Brilliant Violet 711 anti-human CD73, APC/Cyanine7 anti-human CD39 (all from Biolegend), Brilliant Violet 650 anti-human IL-10 (BD Biosciences), and PE-Cyanine7 anti-human TGF-β1 (eBioscience).

### Intracellular Cytokine Staining

Intracellular staining for IL-10 and TGF-β1 was performed on cells stimulated for 5 h with 2 µl/ml cell activation cocktail (with Brefeldin A) (Biolegend) at 37°C and 5% CO_2_. After staining of surface markers, cells were fixed and made permeable with BD Cytofix/Cytoperm solution and Perm/Wash solution in accordance with the instructions of the manufacturer (BD Biosciences).

### Cell Isolation

PB and SF mononuclear cells were isolated using density gradient centrifugation with Ficoll–Paque (Lymphocyte Separation Media; PAA Laboratories). Before density gradient centrifugation, SF was incubated for 30 min at 37°C with 40 µg/ml of hyaluronidase (Sigma-Aldrich). Purification of cell subsets was performed using magnetic cell sorting and confirmed with flow cytometry in accordance with the instructions of the manufacturer. First, CD4^+^ T cells (>93% pure) were isolated by magnetic bead sorting using the CD4 T Cell Isolation Kit (Biolegend). The isolated CD4^+^ T cells were sorted into Teff cells and different Treg subsets using flow cytometry. CD4^+^CD25^−^ T cells were sorted to more than 97% purity using PE anti-human CD4 antibody (Biolegend) and PE-CF594 anti-human CD25 antibody (BD Biosciences). Memory Treg cells were sorted to more than 97% purity as CD4^+^CD127^lo/−^CD25^hi^CD45RA^−^ using Abs including PE anti-human CD4, PE-CF594 anti-human CD25, Alexa Fluor 488 anti-human CD127, and PerCP/Cyanine5.5 anti-human CD45RA (all from Biolegend). Alexa Fluor 647 anti-human CXCR3 (Biolegend) was used for sorting of Th1-like Treg cells. Cells were sorted with a FACS Aria II (BD Biosciences).

### Suppression Assay

Freshly purified T cells (CD4^+^CD25^−^ T cells) were resuspended in phosphate-buffered saline (PBS, with 0.1% bull serum albumin) at a concentration of 2 × 10^6^ cells/ml and incubated for 7 min at 37°C with 5,6-carboxyfluorescein diacetate succinimidylester (CFSE, Sigma-Aldrich, final concentration: 1 μM). Then, CFSE-labeled, sorted 5 × 10^4^ CD4^+^CD25^−^ T cells were used as responders and co-cultured with 5×10^4^ sorted Th1-like Treg cells. These cells were stimulated in round-bottomed 96-well plates with 1 × 10^5^ Treg Suppression Inspector (Miltenyi Biotec). Cellular proliferation was assayed after 5 days using flow cytometry.

### Statistical Analysis

All experimental data were statistically analyzed using GraphPad 5.05. For normally distributed data, the F test was used for comparisons between multiple groups, the SNK-Q test was used for comparisons between two groups, and the Pearson correlation analysis was used for correlation between two groups. For data that did not conform to the normal distribution or variance heterogeneity, the Kruskal–Walls H test was applied for comparison between multiple groups, the Mann–Whitney U test was used for comparison between two groups, and Spearman rank correlation analysis was used for correlation analysis. For all tests, a two-sided *p*-value <0.05 was considered statistically significant.

## Results

### Treg Cells Were Increased in RA

**Figure 1:**
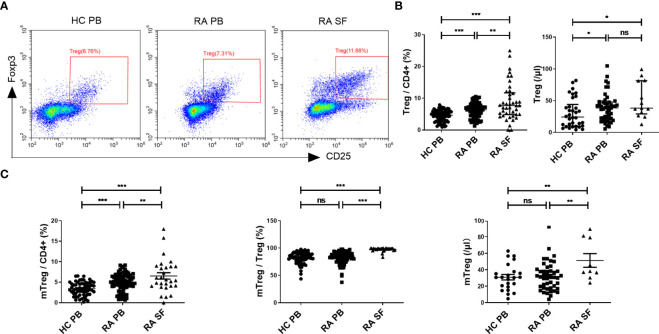
The comparation of Treg cell levels among HC PB, RA PB, and RA SF. **(A)** Flow cytometric dot-plots of Treg cells in HC PB, RA PB, and RA SF. **(B)** Cumulative data of Treg cells in HC PB, RA PB, and RA SF. **(C)** Cumulative data of mTreg cells in HC PB, RA PB, and RA SF. Data are from 86 RA patients, 76 healthy donors and 42 SF samples. *p <0.05, **p <0.01, ***p <0.001, ns, not statistically significant.

In our study, we first examined the changes in the proportion and absolute number of Treg cells (**Figure 1A**) in the PB and SF of RA patients. The median proportion of Treg cells (in CD4^+^ cells) in PB and SF of RA patients was 6.38% [interquartile range (IQR) 4.43–7.75%] and 7.71% (IQR 4.84–11.68%), respectively, and the median in PB of HC was 4.66% (IQR 2.91–5.90%). The pairwise comparison among these three groups shows a statistical difference (**Figure 1B**). The absolute number of Treg cells in the PB and SF of RA patients was significantly higher than that in the PB of HC, while there was no statistical difference between the PB and SF of RA patients (**Figure 1B**). The proportion of memory Treg (mTreg) cells (in CD4^+^ cells) in RA PB, RA SF, and HC PB was 4.96 (IQR 3.72–6.48)%, 5.51 (IQR 4.05–8.84)%, and 3.36 (IQR 2.04–4.79)%, respectively. Similar to the proportion of Treg cells, the pairwise comparison among these three groups shows a statistical difference (RA SF > RA PB > HC PB) (**Figure 1C**). In RA PB, RA SF, and HC PB, the proportion of mTreg cells in Treg cells was 83.37 (IQR 76.68–89.44)%, 98.68 (IQR 97.10–99.31)%, and 83.30 (IQR 77.30–90.17)%, respectively. The proportion of mTreg cells (in Treg cells) and absolute number of mTreg in SF were significantly higher than those in RA PB and HC PB (**Figure 1C**).

### Th1-Like Treg Cells Were Significantly Increased in the SF of RA Patients

**Figure 2:**
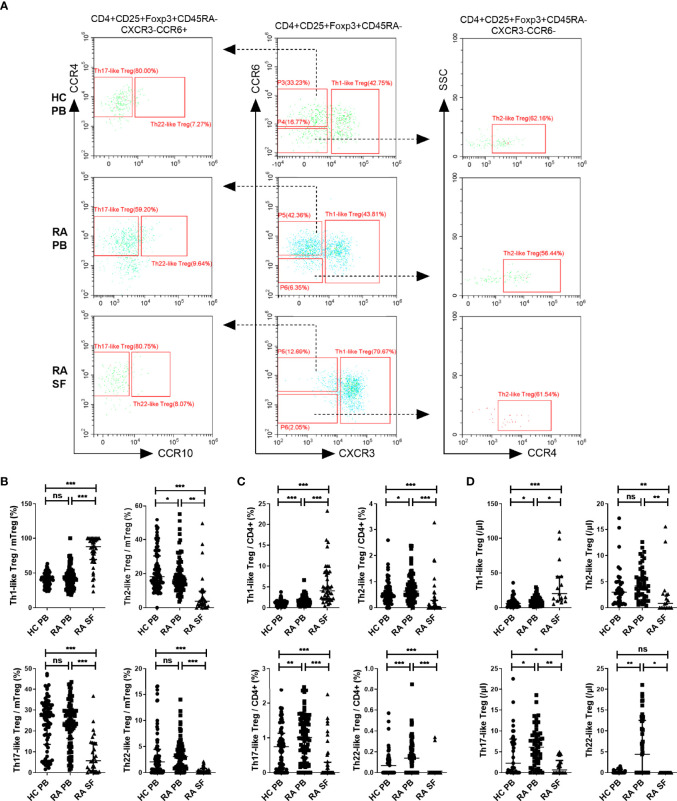
The comparation of different Th-like Treg subsets among HC PB, RA PB, and RA SF. **(A)** Flow cytometric dot-plots and gating strategies of different Th-like Treg cells in HC PB, RA PB, and RA SF. **(B–D)** Cumulative data of different Th-like Treg cells in HC PB, RA PB, and RA SF. Data are from 86 RA patients, 76 healthy donors and 42 SF samples. *p <0.05, **p <0.01, ***p <0.001, ns, not statistically significant.

We next compared the proportion and number of different Th-like Treg cell subsets between RA patients and HC. Based on the differential expression of the chemokine receptors CXCR3, CCR4, CCR6, and CCR10, four subsets of CD4^+^ CD25^+^ FoxP3^+^ CD45RA^−^ memory Treg cells can be defined: Th1-like cells (CXCR3^+^), Th2-like cells (CXCR3^−^CCR6^−^CCR4^+^), Th17-like Treg cells (CXCR3^−^CCR6^+^ CCR4^+^ CCR10^−^), and Th22-like Treg cells (CXCR3^−^CCR6^+^ CCR4^+^ CCR10^+^) (**Figure 2A**). The proportions and absolute counts of the four Th-like Treg cell subsets in HC PB, RA PB, and RA SF are shown in **Tables 2** and **3**. The proportions of Th1-like, Th17-like, and Th22-like Treg cell subsets, but not Th2-like Treg cells, in mTreg from RA PB were similar to those from HC PB (**Figure 2B**). Furthermore, the proportions of all four Th-like Treg cell subsets in CD4^+^ cells from RA PB were higher than those from HC PB (**Figure 2C**). The absolute counts of Th1-like, Th17-like, and Th22-like Treg cells, but not Th2-like Treg cells, were higher than those of HC (**Figure 2D**). In the SF of RA patients, the proportion and absolute number of Th1-like Treg cells were significantly higher than those in the PB of RA patients and HC, while the proportions and absolute numbers of the other three Th-like Treg cell subsets were reduced (**Figures 2B-D** **)**.

**Table 2:** The proportion of different Th-like Tregs in HC PB, RA PB, and RA SF.

Treg subsets	HC PB (n = 76)	RA PB (n = 86)	RA SF (n = 42)
Th1-like Treg/mTreg (%)	40.96 (33.36,46.30)^a^	41.19 (31.39,50.49)^a^	87.80 (69.24,99.61)^a^
Th2-like Treg/mTreg (%)	18.28 (13.47,30.43)	16.44 (11.72,22.23)	3.81 (1.52,9.71)
Th17-like Treg/mTreg (%)	26.87 (13.52,32.23)	24.15 (16.38,29.62)	5.71 (0.95,14.34)
Th22-like Treg/mTreg (%)	2.07 (0.54,4.46)	3.05 (1.11,4.89)	0.30 (0,0.80)
Th1-like Treg/CD4^+^ (%)	1.16 (0.57–1.46)^b^	1.47 (0.91–2.20)^b^	4.05 (2.20–8.54)^b^
Th2-like Treg/CD4^+^ (%)	0.28 (0.31–0.74)	0.64 (0.40–0.92)	0 (0–0.29)
Th17-like Treg/CD4^+^ (%)	0.75 (0.14–1.13)	1.01 (0.43–1.53)	0 (0–0.30)
Th22-like Treg/CD4^+^ (%)	0 (0–0.12)	0 (0–0.14)	0

All values are given as Median (IQR).

a p ^<^0.05 for Th1-like Treg vs. the other three Th-like Treg in mTreg (%);

b p ^<^0.05 for Th1-like Treg vs. the other three Th-like Treg in CD4^+^ T cells (%).

**Table 3:** Absolute counts of different Th-like Treg cells in HC PB, RA PB, and RA SF.

Treg subsets	HC PB (n = 34)	RA PB (n = 48)	RA SF (n = 13)
Th1-like Treg (/μl)	6.09 (1.78–10.90)	8.4 (4.27–14.49)	20.39 (9.90–45.18)
Th2-like Treg (/μl)	2.99 (1.11–5.02)	4.18 (2.21–6.43)	0.82 (0–2.66)
Th17-like Treg (/μl)	2.26 (0–8.03)	6.10 (2.64–9.14)	0.67 (0–2.92)
Th22-like Treg (/μl)	0 (0–0.75)	4.38 (0–12.53)	0

All values are given as Median (IQR).

### The Proportions of Treg Cells and Th1-Like Treg Cells in the SF of RA Patients Were Negatively Correlated With Disease Activity

**Figure 3:**
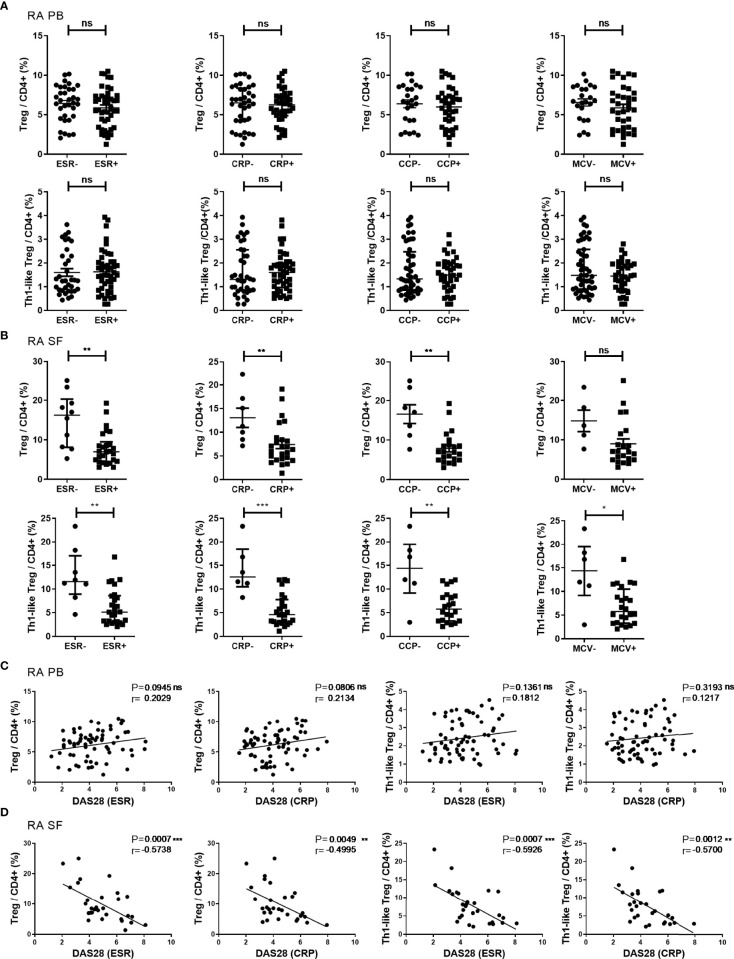
The proportion of Treg cells and their subsets in different disease states and their correlations. **(A, B)** Comparison of the proportions of Treg cells and Th1-like Treg cells in RA PB **(A)** and RA SF **(B)** in subgroups based on clinical indicators. **(C, D)** Correlation analysis between the proportions of Treg cells or Th1-like Treg cells and DAS28 in RA PB **(C)** and RA SF **(D)**. Data are from 78 RA patients and 36 SF samples. *p <0.05, **p <0.01, ***p <0.001, ns, not statistically significant.

Next, RA patients were grouped by the status of ESR, CRP, ACPA, and disease activity, and the proportion of different subsets of Treg cells among different groups was compared. There was no significant difference in the proportions of Treg cells and Th1-like Treg in the PB of RA patients in the different groups (**Figure 3A**). In the SF of RA patients, the proportions of Treg cells and Th1-like Treg cells were significantly lower in the groups with elevated ESR (ESR >20 mm/h) or CRP (CRP ≥0.8 mg/dl) and in the groups positive for anti-CCP antibody (anti-CCP antibody ≥25 RU/ml). Furthermore, Th1-like Treg cells were also significantly lower in the groups positive for anti-MCV antibody (anti-MCV antibody ≥20 U/ml) (**Figure 3B**). The proportion of other Th-like Treg cells showed no difference between different groups (data not shown). In our study, correlation analysis was conducted between the levels of Treg cell subsets and ESR, CRP, and DAS28 levels. The proportion of Treg cells or Th-like Treg cells in the PB of RA patients showed no significant correlation with DAS28 (**Figure 3C**). However, the proportions of Treg cells and Th1-like Treg cells in the SF of RA patients were negatively correlated with disease activity (**Figure 3D**), but there was no significant correlation between the proportions of other Th-like Treg cells and disease activity (data not shown).

### The Expression Levels of CD73 and TGF-β1 in Th1-Like Treg Cells From RA SF Were Reduced

**Figure 4:**
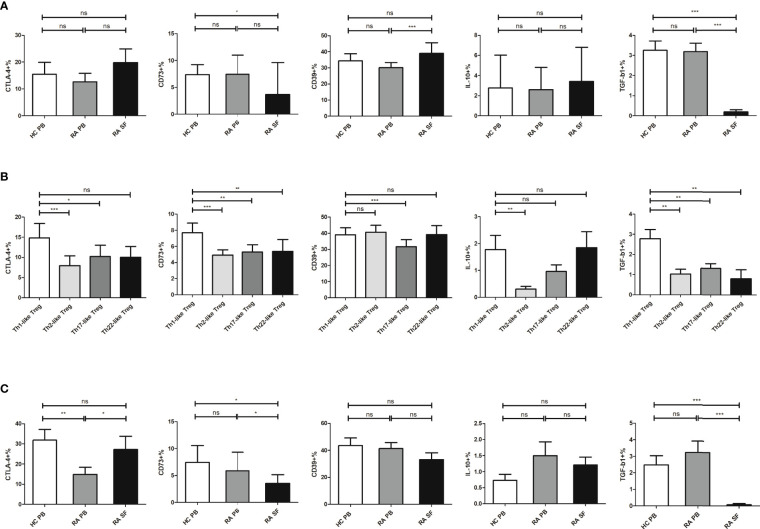
The expression level of different molecules in Treg and its subsets. **(A)** Comparison of the expression levels of CTLA-4, CD73, CD39, IL-10, and TGF-β1 in Treg cells from HC PB, RA PB, and RA SF. **(B)** Comparison of the expression levels of CTLA-4, CD73, CD39, IL-10, and TGF-β1 in different Th-like Treg cells from RA PB. **(C)** Comparison of the expression levels of CTLA-4, CD73, CD39, IL-10, and TGF-β1 in Th1-like Treg cells from HC PB, RA PB, and RA SF. Data are from 48 RA patients, 39 healthy donors and 16 SF samples. *p <0.05, **p <0.01, ***p <0.001, ns, not statistically significant.

Next, we compared the expression levels of functional molecules, including CTLA-4, CD73, CD39, IL-10, and TGF-β1, in Treg cells from RA patients and HC. The expression levels of these functional molecules in RA PB were not significantly different from those in HC PB. The expression level of CD39 in Treg cells from RA SF was higher than that of RA PB, but the expression levels of CD73 and TGF-β1 were significantly decreased (**Figure 4A**). The expression levels of functional molecules of different Th-like Treg cell subsets in RA PB were analyzed. Compared with the other three groups of Th-like Treg cell subsets, the expression levels of inhibitory functional molecules of Th1-like Treg cells in RA PB was higher (**Figure 4B**). Then we focused on the comparison of Th1-like Treg cells from different samples and found that the expression levels of TGF-β1 and CD73 in Th1-like Treg cells from RA SF were significantly lower than those of RA PB and HC PB (**Figure 4C**). There were no significant differences in the expression levels of CD39 and IL-10 among all groups (**Figure 4C**).

### Th1-Like Treg Cells From RA SF Did Not Inhibit Teff Cell Proliferation *In Vitro*

**Figure 5:**
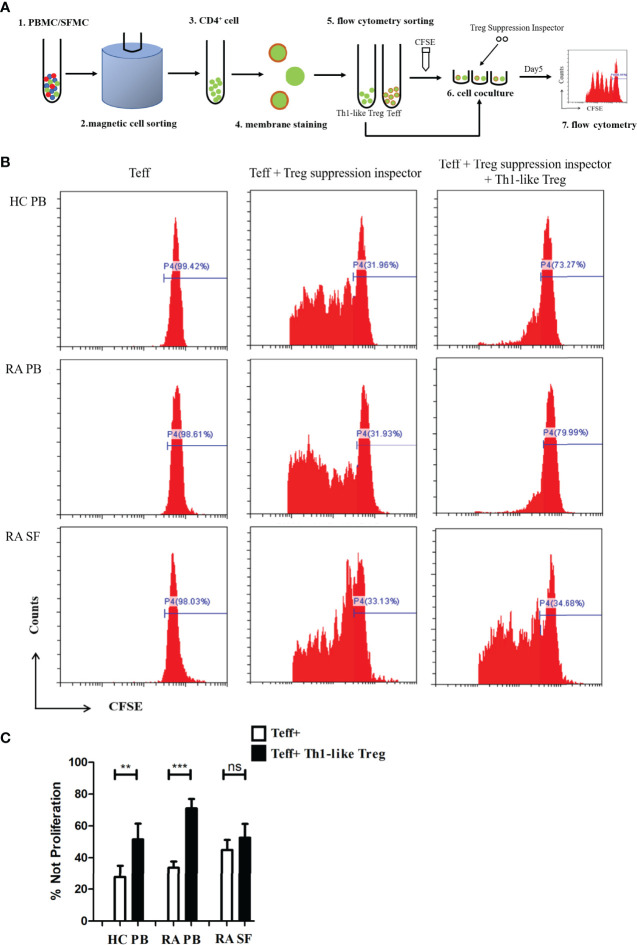
Th1-like Treg cell suppression assay *in vitro*. **(A)** Schematic of the Treg *in vitro* suppression assay. **(B)** Histograms of Teff cell proliferation from HC PB, RA PB, and RA SF under different conditions. **(C)** Comparison of the suppression of Teff cell proliferation by Th1-like Treg cells from HC PB, RA PB, and RA SF. Data are from 11 RA patients, 4 healthy donors and 5 SF samples. **p <0.01, ***p <0.001, ns, not statistically significant.

To further investigate the function of Th1-like Treg cells, we selected CD4^+^CD25^−^ Teff cells and CD4^+^CD25^hi^CD127^low/−^ CD45RA^−^CXCR3^+^ Th1-like Treg cells using magnetic bead sorting and flow cytometry, and carried out an *in vitro* suppression assay (**Figure 5A**). Teff cell proliferation was significantly inhibited when the cells were co-cultured with autologous Th1-like Treg cells from RA PB or HC PB (**Figures 5B, C** **)**. However, Th1-like Treg cells from RA SF could not inhibit the proliferation of Teff cells (**Figures 5B, C** **)**, suggesting that Th1-like Treg cells from RA SF were defective in inhibitory function.

## Discussion

Previous studies suggest that Treg cells are involved in the pathogenesis of RA, and an insufficient number or impaired function of Treg cells is one of the important pathogenic mechanisms of RA. However, conclusions from the studies focused on the changes in the number of Treg cells in RA are still controversial. There are several reasons for these discrepancies in the literature. First, various biomarkers were used to identify Treg cells in different experiments; in many early studies, only surface markers (such as CD4 and CD25) were used to identify Treg cells. Secondly, the heterogeneity of the disease courses, activities, and treatments of RA patients, could be a contributing factor. Finally, the heterogeneity of Treg cell subsets may change in RA. We first analyzed the overall proportion and absolute number of CD4^+^CD25^+^Foxp3^+^ Treg cells and found that the proportions of Treg cells in RA PB and RA SF were higher than that in HC PB, and the proportion of Treg cells in RA SF was higher than that in RA PB. Similar to Treg, the proportion and absolute count of mTreg in RA SF were much higher than those in RA PB and HC PB.

Secondly, we analyzed four Th-like Treg cell subsets. The most striking finding was that in both RA PB and RA SF, the proportion of Th1-like Treg cells significantly increased, especially in SF, accounting for 87.8% of memory Treg cells. This suggests that Th1-like Treg cells are the dominant Treg subsets in RA SF. Studies have shown that the expression of various inflammatory chemokines and chemokine receptors is upregulated in the SF and synovial tissue of RA patients, among which the expression levels of CXCL10 and CXCL11 ligands of CXCR3 in SF are significantly increased (28). Consistent with these previous findings, we found that the proportion and absolute number of Th1-like Treg cells expressing CXCR3 were significantly increased in RA SF. Although the proportion and number of Treg cells in the SF were increased, the inflammatory response could not be effectively controlled, suggesting that Treg cells have a deficiency in their inhibitory function.

Previous studies have also reported divergent views on the correlation between the number of Treg cells and disease activity indicators (such as DAS28) (29–31). These discrepancies may be related to the different phenotypic markers used to detect Treg cells. According to one study, the proportion of CD4^+^CD25^+^ T cells or CD4^+^CD25^+^CD127^low/−^ T cells in PB of RA patients was not correlated with DAS28, but there was a negative relationship between the proportion of CD4^+^CD25^hi^CD127 ^low/−^ T cells and DAS28 (32). In our work, there was no correlation between the proportion of Treg cells or their subgroups and DAS28. However, in RA SF, the proportion of both Treg and Th1-like Treg was negatively correlated with DAS28. This indicates that the higher the disease activity of RA patients, the lower the proportion of Treg cells and Th1-like Treg cells in SF, suggesting that Treg cells still play a partial protective role in RA and serve as an indicator of RA disease activity.

RF (33) and ACPA (34) can appear before the onset of RA and play a certain predictive role in the occurrence and development of RA. Further, RF and ACPA are associated with joint damage and extra-articular symptoms of RA (35, 36). Anti-MCV antibodies could also reflect disease activity (37) and predict radiological damage (38). Furthermore, AKA had a considerably high specificity for RA diagnosis (39). In our study, we not only explored the relationship between the proportion of Treg cells and their subsets and DAS28 [non-specific inflammatory indexes (ESR and CRP)] but also analyzed the relationship between Treg cells and RF, ACPA, or AKA status. The proportion of Treg cells and Th1-like Treg cells in the SF in the positive group of ESR, CRP, or anti-CCP antibody was significantly lower than that in the negative group. These data indicate that at the site of inflammation, when the disease is active or ACPA is positive, the proportion of Treg cells and Th1-like Treg cells decreases, correlating with increased inflammation and exacerbation of RA.

So far, changes in the function of Treg cells in RA have been disputed (13, 14). Differences in Treg function may be closely related to the heterogeneity of Treg cells. In our study, we observed differences in the expression levels of functional molecules of different Th-like Treg cells in the PB of RA patients. Th1-like Treg cells express higher levels of CD73 and TGF-β1 compared to other Th-like Treg subsets, suggesting that Th1-like Treg cells have stronger inhibitory functions than other cell subsets. Since Th1-like Treg cells are the dominant Treg subsets in RA SF (87.8% of memory Treg cells), while the proportions of other Th-like Treg cells are relatively very low, there may be obvious experimental errors in the detection of functional molecules among other Th-like Treg subsets. Therefore, instead of presenting these results, we compared the expression levels of functional molecules in Th1-like Treg cells from HC PB, RA PB, and RA SF. In RA SF, the expression levels of CD73 and TGF-β1 in Th1-like Treg cells were significantly decreased, indicating that Th1-like Treg cells in SF may have a deficiency in inhibitory function, which may be a reason for the failed control of local inflammation in RA joints. Our *in vitro* suppression assay showed that Th1-like Treg cells in HC PB and RA PB could effectively inhibit the proliferation of Teff cells, while Th1-like Treg cells in RA SF could not inhibit the proliferation of Teff cells, further indicating that the inhibitory function of Th1-like Treg cells in RA SF is defective. However, it remains to be determined how Th1-like Treg cells lose their inhibitory function, how Treg cells affect apoptosis and effector molecule production of Teff cells and other inflammatory cells, and the molecular mechanism underlying this pathology. Thus, further studies are needed.

There are some limitations to our work. Although we analyzed Treg proportion in patients with different treatment backgrounds, there was no significant difference in the proportion of different Treg subsets between different patient groups (data not shown), which may be explained by the small sample size after grouping, disease heterogeneity, and combination administration. However, the possible effects of therapeutic drugs on Treg cell levels during blood sampling cannot be ignored. Thus, further investigations with an expanded sample size, detailed medication information, and follow-up observation are needed to confirm and extend the current findings. Additionally, as Treg may secrete pro-inflammatory cytokines such as IL-17 and undergo phenotypic transformation under certain conditions (40), this will be the focus of our next work, to have a more comprehensive understanding of Treg subsets and their fate in RA.

In conclusion, Th1-like Treg cells are the predominant Treg cell subset in RA SF, but their suppressive function is defective in RA. Improving the inhibitory function of Th1-like Treg cells may control the local inflammatory response in joints and provide a new strategy for RA-targeted therapy.

## Data Availability Statement

The original contributions presented in the study are included in the article/supplementary material. Further inquiries can be directed to the corresponding authors.

## Ethics Statement

The studies involving human participants were reviewed and approved by the Ethics Committee of Xijing Hospital. The patients/participants provided their written informed consent to participate in this study.

## Author Contributions

RZ, JM, and KZ performed most of the experiments and wrote the manuscript. BZ was in charge of the recruitment of patients and clinical data collection. XL and HS participated in the experiments. All authors listed have made a substantial, direct, and intellectual contribution to the work and approved it for publication.

## Funding

This work was supported by grants from the National Key Research and Development Program of China [grant number 2017YFC0909002] and the National Natural Science Foundation of China [grant number 81801599].

## Conflict of Interest

The authors declare that the research was conducted in the absence of any commercial or financial relationships that could be construed as a potential conflict of interest.

## Publisher’s Note

All claims expressed in this article are solely those of the authors and do not necessarily represent those of their affiliated organizations, or those of the publisher, the editors and the reviewers. Any product that may be evaluated in this article, or claim that may be made by its manufacturer, is not guaranteed or endorsed by the publisher.
